# A Systematic Review of Interventions Addressing Adherence to Anti-Diabetic Medications in Patients with Type 2 Diabetes—Components of Interventions

**DOI:** 10.1371/journal.pone.0128581

**Published:** 2015-06-08

**Authors:** Sujata Sapkota, Jo-anne E. Brien, Jerry R. Greenfield, Parisa Aslani

**Affiliations:** 1 Faculty of Pharmacy, The University of Sydney, Sydney, NSW, Australia; 2 Department of Endocrinology, St. Vincent Hospital, Sydney, NSW, Australia; Karolinska Institutet, ITALY

## Abstract

**Background:**

Poor adherence to anti-diabetic medications contributes to suboptimal glycaemic control in patients with type 2 diabetes (T2D). A range of interventions have been developed to promote anti-diabetic medication adherence. However, there has been very little focus on the characteristics of these interventions and how effectively they address factors that predict non-adherence. In this systematic review we assessed the characteristics of interventions that aimed to promote adherence to anti-diabetic medications.

**Method:**

Using appropriate search terms in Medline, Embase, CINAHL, International Pharmaceutical Abstracts (IPA), PUBmed, and PsychINFO (years 2000–2013), we identified 52 studies which met the inclusion criteria.

**Results:**

Forty-nine studies consisted of patient-level interventions, two provider-level interventions, and one consisted of both. Interventions were classified as educational (n = 7), behavioural (n = 3), affective, economic (n = 3) or multifaceted (a combination of the above; n = 40). One study consisted of two interventions. The review found that multifaceted interventions, addressing several non-adherence factors, were comparatively more effective in improving medication adherence and glycaemic target in patients with T2D than single strategies. However, interventions with similar components and those addressing similar non-adherence factors demonstrated mixed results, making it difficult to conclude on effective intervention strategies to promote adherence. Educational strategies have remained the most popular intervention strategy, followed by behavioural, with affective components becoming more common in recent years. Most of the interventions addressed patient-related (n = 35), condition-related (n = 31), and therapy-related (n = 20) factors as defined by the World Health Organization, while fewer addressed health care system (n = 5) and socio-economic-related factors (n = 13).

**Conclusion:**

There is a noticeable shift in the literature from using single to multifaceted intervention strategies addressing a range of factors impacting adherence to medications. However, research limitations, such as limited use of standardized methods and tools to measure adherence, lack of individually tailored adherence promoting strategies and variability in the interventions developed, reduce the ability to generalize the findings of the studies reviewed. Furthermore, this review highlights the need to develop multifaceted interventions which can be tailored to the individual patient’s needs over the duration of their diabetes management.

## Introduction

Diabetes, characterized by hyperglycaemia, is one of the major chronic conditions that impacts a significant proportion of the population worldwide [1]. Diabetes is projected to be the 7th leading cause of death in 2030 [2]; with type 2 diabetes (T2D) accounting for most of the cases [2].

Management of T2D involves weight loss, via reductions in caloric intake and increases in physical activity, oral anti-diabetic agents and/or insulin [3, 4]. Non-pharmacological measures remain one of the key management recommendations, and are usually the starting point for patients diagnosed with T2D [3, 4]. Different classes of anti-diabetic agents are available for treatment, and dual, followed by combination therapy is recommended if mono-therapy is insufficient. Patients may ultimately require insulin replacement therapy to adequately manage their diabetes [4].

While adequate management of diabetes is extremely important for the prevention or delay of complications arising from poorly controlled blood sugar levels, the ‘chronic’ nature of the disease, lifelong requirement for medications, requirement for changes in lifestyle, the need to cope with social, cultural and psychological distress that may occur with the disease, and the clinical manifestations and associated complications, make the management of diabetes very complex. There is also a need to individually tailor the therapy for diabetic patients depending upon their age, demographic variability, co-morbidities and risk of developing complications [3].

Amidst this complexity, remaining adherent to treatment recommendations, such as home glucose monitoring, adjustment of food intake, administration of medication(s), regular physical exercise, foot care and regular medical visits [5] may be a challenge [6]. Adherence to medications has been recognized as key for optimally controlled diabetes [7, 8] in T2D patients.

Many factors affect adherence to treatment in diabetes such as disease and treatment characteristics and complexity, age, gender, self-esteem, stress, depression, quality of the relationship between patients and health care providers, social support, and patients’ ability to remain adherent amidst changing circumstances in their daily life [5]. The World Health Organization (WHO) has recognized five factors which influence adherence to all medications. They are health system related factors, socio-economic factors, condition related factors, therapy related factors and patient related factors [5]. Adherence is therefore a multifactorial behaviour and solutions require acknowledgment of the range of factors.

Studies have shown that medication adherence rates among patients with T2D is not optimal and is comparable to those with other chronic diseases [9]. Adherence to oral diabetes medications in T2D patients is 36 to 93% and to insulin is around 60% [10]. Treatment non-adherence in diabetes is well recognised, and interventions to promote adherence, improve glycaemic control, self-care behaviours and other key outcomes have been designed and implemented in T2D patients. Several reviews and meta-analyses published over the last decade have addressed these interventions in T2D patients [11–23].

Some reviews have focused on the wider aspect of treatment adherence in diabetes, incorporating not just adherence to anti-diabetic medications but treatment adherence as a whole [14, 15, 20]. Where adherence to medications are specifically discussed [12, 17, 22, 23], the reviews have included studies that analysed adherence to a range of medications taken by T2D patients, rather than being specific to anti-diabetic medications [12]. Only a limited number of reviews have specifically evaluated interventions assessing adherence to anti-diabetic medications [17, 22, 23]. However, there has been very little focus on the characteristics of these interventions and how well they address factors that impact non-adherence.

Coincidentally, a review [24] examining interventions to improve medication adherence in patients with T2D was published at the same time as the current review was being finalized. However, the review has broad inclusion criteria and does not focus specifically on adherence to anti-diabetic medications. Furthermore, there was no in-depth categorization of the intervention components or an exploration of non-adherence factors addressed by the interventions. These are key gaps identified in the existing reviews of the literature. Therefore, the current review aims to assess the characteristics of the interventions, focusing on their components and evaluating how they address the factors that contribute to non-adherence to anti-diabetic medications. The specific objectives are to:

- identify and categorise the intervention components which have led to improved medication adherence to anti-diabetic medications;
- determine the WHO non-adherence factors that the interventions have addressed; and
- evaluate changes in intervention designs and implementation techniques over time.

## Methods

### Literature search

A review of the literature was conducted to identify research articles that have evaluated the impact of interventions on adherence to anti-diabetic medications in T2D patients. Studies were searched in the following databases: Medline, Embase, CINAHL, International Pharmaceutical Abstracts (IPA), PUBmed, and PsychINFO. Each database was searched using the appropriate terms for medication adherence (concept 1), type 2 diabetes/ anti- diabetic medications (concept 2) and intervention studies (concept 3). Key words/ terms to denote these concepts were used and then combined using ‘and’ operator (concept 1 and concept 2 and concept 3) (S1 Fig). The search strategy was limited to articles published from January 2000 to April 2013 (inclusive) and published in English. The references of relevant publications (all studies included in this review and relevant systematic reviews [12, 14, 15, 17, 20, 21, 23] were hand searched to find additional studies that met the inclusion criteria (S1 Table). The search strategy as well as the study inclusion and exclusion criteria have also been illustrated previously [25].

### Data extraction and analysis

For each study, the “intervention” and its characteristics were evaluated in-depth. The intervention components were summarised and the impact of each intervention was recorded. The interventionist(s), provision of training to the interventionist(s) before delivering the intervention, and the method of evaluating the delivery of the intervention were assessed. The interventions were categorised as recommended by Roter *et al*. [26]. Further information about the study characteristics can be found elsewhere [25].

### Operational definitions

For the purposes of this review, the following operational definitions were used:

- The term ‘medication adherence/ adherence’ has been used throughout the review to indicate the extent to which individuals take their medication, despite the alternative terms used in the studies included in the review.
- Types of Interventions
- Interventions have been classified into Educational, Behavioural and Affective, where appropriate, following the classification used by Roter *et al* [26]. Two additional categories were also used:
  - Educational: “*pedagogic interventions*, *verbal or written*, *with a knowledge based emphasis designed to convey information*. *Specific strategies included one-to-one and group teaching*, *the use of written and audio visual materials*, *mailed materials*, *and telephone instructions”* [26].
  - Behavioural: “*interventions that were designed to change compliance by targeting*, *shaping*, *or reinforcing specific behavioural patterns*. *This included strategies such as skill building and practice activities*, *behavioural modelling and contracting*, *packaging and dosage modifications or tailoring*, *rewards*, *and both mail and telephone reminders”* [26].
  - Affective: “*strategies that attempted to influence adherence through appeals to feelings and emotions or social relationships and social supports*. *Included were family support*, *counselling*, *and supportive home visits”* [26].
  - Economic: interventions that dealt with economic or cost related issues pertaining to medications.
  - Multifaceted: interventions that had components that could be categorized into more than one of the above categories.

## Results

### 1. Study selection

The literature search identified 6,662 citations. A total of 230 articles appeared to meet the review inclusion criteria and were retrieved in full text. However, only 49 studies actually met the study criteria and were selected for review. Three more studies were identified from hand searching. Thus, a total of 52 studies [27–78] were included [25] (S2 Fig).

### 2. Study characteristics

The majority (57.7%) of the studies were conducted in the USA and most (n = 38) were published in the last 5 years. Approximately half of the studies were ‘randomized controlled’ [28, 29, 31, 34, 35, 37, 39, 43, 47–50, 56, 59, 60, 62–64, 66, 67, 69, 72, 75–77]. Participant inclusion criteria varied across the studies in terms of age, HbA1c value, duration since diagnosis, sample size and duration of study. Most of the interventions were carried in community settings (67.3%), and most were conducted for a maximum of 1 year, with the impact of a majority of the interventions being assessed for a duration of 6 months.

In addition to assessing anti-diabetic medication adherence as a primary (73.1%) [27–33, 35–38, 40, 42–50, 53–57, 59, 61, 64, 68–72, 74–77] or a secondary (26.9%) outcome, the studies assessed a range of other clinical or patient specific outcomes. The study characteristics have been discussed in detail previously [24].

The most widely used method for measuring anti-diabetic medication adherence was self-report, and the commonest tool reported was the Summary of Diabetes Self-care Activities (SDSCA) questionnaire. Overall, the methods for measuring anti-diabetic medication adherence varied across the studies, and the implications have been discussed elsewhere [25].

### 3. Interventions and their characteristics

#### 3.1. Classifying the interventions

Forty nine manuscripts dealt with interventions directed at the patient [27–29, 31, 33–51, 53–78], two at the healthcare provider [30, 32], and one at both [52] (Table 1). Most (n = 40) [27–30, 32–43, 47, 48, 50–56, 58, 60–62, 65–67, 69, 70, 72–76, 78] were complex interventions with more than one component, and have been categorized as ‘multifaceted’. Others have been classified as educational (n = 7) [44, 45, 49, 59, 63, 68, 71], behavioural (n = 3) [31, 64, 77] or economic (n = 3) [46, 57, 68]. There were no interventions that were solely ‘affective’.

**Table 1:** Intervention types, elements, WHO factors addressed and interventionists.

Study	Type of intervention	Major elements of intervention process	Dimension(s) of WHO adherence factors targeted by the intervention	Interventionist(s)	Were the interventionist(s) trained (for intervention delivery) and intervention assessed?	Was the intervention successful in improving medication adherence?
Hendricks LE, Hendricks RT, 2000 [27]	Multifaceted *[Educational + Behavioural]*	2-hour diabetes self- management skill training classes for all (two comparison groups); Comparison groups varied in terms of follow up duration. One group received monthly telephone follow up, while the other received the follow up at 3-months’ interval over 6-months study duration; Follow up conducted to evaluate the patients’ progress in achieving treatment goals, assess problems, and track the progress of selected outcomes.	Condition related and Patient related	‘*Investigators*’	Not specified	No
Grant RW et al, 2003 [28]	Multifaceted *[Educational + Affective+ Behavioural (*?*)]*	Assessment of adherence barriers and overall adherence rates and identification of discrepancies in medicine prescription &/or medication use by pharmacist; Medicine- specific patient education, & arrangement for social services or nutritional consultations as required; E-mail (in specified format) to the primary care provider identified by the patient summarizing discrepancies and adherence behaviour and offering to help arrange a follow up appointment.	Therapy related, Condition related, Patient related and Health care system related	Pharmacists	Not specified	No
Kim HS, Oh JA, 2003 [29]	Multifaceted *[Educational+ Behavioural]*	Prior to intervention: a Diabetes care booklet & daily log book provided to the patients; Then, continuous education and reinforcement of diet, exercise and medication adjustment recommendations plus frequent self-monitoring of glucose via telephone intervention for 12 weeks	Condition related, Therapy related and Patient related	Nursing PhD student	Not specified	No
Maddigan SL et al, 2004 [30]	“Provider Level Intervention” Multifaceted *[Educational + Behavioural]*	Diabetes outreach service, consisting of a team of specialist physicians, nurse educators, dieticians and pharmacists, delivered targeted educational messages, to the physicians in smaller groups, with an aim to promote the vascular health [monthly visits for 6 months]. Specific components of the intervention included: Small group discussions of real and theoretical cases related to risk factors, delivered by well- known and respected specialists; one-on-one academic detailing by a trained pharmacist; and a referral service for a limited number of patients; In services provided for allied health care professions in groups of 8 to 30, which focused on the importance and management of risk factors; and, Public lectures focusing on self-management of risk factors.	Health care system related	Intervention to the providers were delivered by “Specialist physicians, Pharmacist, nurse educators and dietician (team)”. The providers then provided their service to the patients	Not specified for the interventionists providing intervention to the intervention region (a professional team was involved). The providers of the regions were trained and assessed as part of intervention.	No
Rosen MI et al, 2004 [31]	Behavioural	Patients provided with cue dose training and with smart caps (MEMS caps) that displayed the number of hours since the last bottle opening and could be programmed to beep at predetermined times. Patients were asked to consider cues to remind them to take their medicines. MEMS data provided to the care givers each month, who were urged to use and discuss the data with patients during scheduled follow up.	Patient related and Health care system related	Cue dose training were conducted by ‘*bachelor level research assistants'*	Not specified	Yes
Schectman JM et al, 2004 [32]	Provider level intervention Multifaceted *[Educational + Behavioural]*	A 2 page patient feed- back report provided to the physicians, which consisted of patient data: lab reports and summary refill-based adherence data for diabetes medications, including percentage adherence (oral agents only) and average daily dose of medication (oral agents and insulin). The providers in the intervention group attended a 30-minute educational session, which reviewed and dealt with feedback reports, techniques for patient adherence assessment, barrier evaluation, and intervention strategies.	Health care system related	Physicians received the intervention in the form of feedback and education. Who delivered the education is not specified.	The intervention received by patients involved trained physicians and assessment of the process as part of intervention. Not specified for the providers of 'educational session' to the physicians.	Yes
Wermeille J et al, 2004 [33]	Multifaceted *[Educational+ Behavioural]*	Identification of any potential or actual medication related problems for each patient from medical general practice notes, the community pharmacy Patient Medication Record (PMR) system and a structured patient interview (approx. 30 min); Pharmaceutical care plan (PCP), generated for each patient based on information compiled onto a specially designed patient medication profile; For each pharmaceutical care issue (PCI) identified, a desired output and proposed action documented, the issues peer reviewed and discussed with the patient’s physician. Decisions then taken based on the discussions, and relevant actions delivered to the patient as appropriate. PC issues were divided into 3 categories: drug therapy, monitoring, patient knowledge.	Therapy related and Patient related	Pharmacist	Not specified	No
Odegard PS et al, 2005 [34]	Multifaceted *[Educational+ Behavioural]*	Development of diabetes care plan (based on pharmacist patient and pharmacist provider communication), its individualization and implementation; Regular pharmacist-patient communication on diabetes care progress to make the patient follow on the DCP, modification as needed; Assessment of progress, reactivation of the intensive phase (weekly) for new problems or changes in therapy.	Therapy related and Patient related	Primary care pharmacist	Not specified	No
Keeratiyutawong P et al, 2006 [35]	Multifaceted *[Educational+ Behavioural+ Affective]*	5 educational sessions each of 2 hours conducted in groups of 9–13 patients. The session dealt with: (1) a pathology of diabetes mellitus, cognitive restructuring and goal setting skills; (2) dietary control and communication skills; (3) diabetes medication, and problem solving skills; (4) foot care and self-monitoring; and (5) exercise. Follow-up by telephone call at the 3rd& 5thmonth, to discuss about the subjects diabetes self-management and the problems of self-management practices, and to provide support and reinforcement to the participants to maintain their self-management.	Condition related, Therapy related and Patient related	*'A researcher with experience of caring for Diabetic patients conducted the intervention'*. *'Research assistants helped the researcher facilitate the self-management program'*.	**Training**-Yes (?) (The researcher attended a diabetes self-management program and had experience in conducting participatory action research in promoting self-care for patients. The research assistants were trained to collect questionnaires); **Assessment**- Not specified	No
Kim HS et al, 2006 [36]	Multifaceted *[Educational + Behavioural]*	Patient asked to input their blood glucose level via cellular phone or internet; Continuous education and reinforcement of diet, exercise, medication adjustment, and frequent self- monitoring of blood glucose levels via SMS; Medicine adjustment communicated with the physician.	Condition related and Therapy related	Nurse researcher	Not specified (the researchers followed a protocol)	Yes
Vincent D et al, 2007 [37]	Multifaceted *[Educational + Behavioural + Affective]*	8 weekly 2-hr group sessions, which included didactic content, cooking demonstrations, and group support, with identified cultural components integrated into the sessions. Strategies to foster self-efficacy were incorporated and included skill mastery of self-glucose monitoring, problem solving, and verbal persuasion. The sessions covered disease related issues, like pathophysiology, complications, treatment and self-management strategies; stress/ stress management, heredity and culture. Patients encouraged to bring support person to the sessions.	Condition related, Therapy related, Patient related and **Socio**- economic related	Not specified	Not specified	No
Faridi Z et al, 2008 [38]	Multifaceted *[Behavioural + Educational]*	The intervention used NICHE technology, an interactive informational feedback system using wireless remote technology to provide tailored feedback and reminders, based on patient-specific data, to patients and providers via messages on cellular phones. Patients were given 1- day training on NICHE technology. Patients were required to test their glucose once daily (upon awakening) and wear their pedometers during the day, and to upload data onto the NICHE server once daily. Based on the uploaded data, they received tailored messages via mobile phone.	Patient related	Nurse practitioners	**Training**-Yes(?) ‘*Nurse practitioner attended a comprehensive training sessions in the NICHE system’*. Nothing specified about the overall process though.	No
Quinn CC et al, 2008 [39]	Multifaceted *[Educational + Behavioural]*	WellDoc’s System (WDS), which served as virtual coach for patients and a virtual endocrinologist for HCPs, facilitating the coordination of diabetes care among existing resources used; Personalized real time feedback given by the system in response to patient data uploaded; Educational material emailed to patients after identification of the problem; Guided compliance tool which directed patients to test their BG at optimal times; WDS suggested medication change recommendation to the HCPs.	Condition related and Therapy related	‘*Investigators*’	Not specified	No
Utz SW et al, 2008 [40]	Multifaceted *[Educational + Behavioural + Affective]*	Group DSME consisted of culturally tailored diabetes education in a supportive learning environment; hands on activities; reviewing goals, obstacles and progress, problem- solving, delivered by diabetes educators for 8 weeks, @ 2hr/ week sessions. Adults learning principles were emphasized by using role model and group leaders. Individual DSME focused on: Goal setting & problem solving and review of progress conducted at week 1, 4 and 8 respectively	Condition related, Patient related and **Socio**- economic related	Diabetes educators	Not specified	No
Babomoto KS et al, 2009 [41]	Multifaceted *[Educational+ Behavioural+ Affective]*	CHW’s conducted individual education sessions based on ADA standards, tailored to patients’ requirement as appropriate. Use of culturally appropriate educational materials based on stages of change, with the assessment of patient understanding, knowledge deficit etc; Education, positive reinforcement, and tips on achieving health behaviour goals; Follow up telephone calls to monitor self-management progress, identify barriers / issues and assist in problem solving.	Condition related, Patient related and **Socio**- economic	CHW group: Community health workers (CHW); CM group: Nurses	CHW were **trained**. Nothing specified about the Nurses. **Assessment**- Not specified	No
Clarke A, 2009 [42]	Multifaceted *[Educational + Behavioural]*	Routine educational sessions (2hrs), consisting of presentation of basic diabetes knowledge and skills by diabetes nurse. Follow up session 2 weeks later on advanced knowledge and skill.	Condition related	Diabetes nurse	Not specified	No
Glassgow RE et al, 2009 [43]	Multifaceted *[Educational + Behavioural]*	Two groups, the 'classes' group and 'DVD' group. Classes: Patients attended "taking charge of your diabetes" course comprising of 2 classes, each of 2.5 to 3 hours. The curriculum was focused on 7 areas of diabetes management. Additionally, the instructors facilitated group discussion around each topic by encouraging members to:(1) Self-assess, (2) Share key information, preferably in bullet points(3) Set specific goals in that area. Each member attending class was given the diabetes manual, a comprehensive text written in a workbook format. DVD: Patients only received the DVD, titled ‘Diabetes and balance: my personal roadmap’ which had 7 chapters around 7 self- care areas. Goal-setting and problem-solving approach was similar to the class. Viewers were instructed to pause the DVD and complete an action plan page in an accompanying workbook. Classes + DVD: Patients were mailed the DVD immediately after the second class.	Condition related and Patient related	For DVD: Not applicable. Classes: Nurses, diabetes educators and clinical pharmacists involved as appropriate.	Not specified	No
Kolawole B et al, 2009 [44]	Educational	Diabetes self- management and multidisciplinary education program conducted by Diabetes Association of Nigeria (DAN)	Condition related and Therapy related	‘*Relevant health professional and diabetes educators attended in turns & organized 1–2 hours session*’	Not specified (The program was conducted according to a protocol)	Yes
Mullan RJ et al, 2009 [45]	Educational	‘Diabetes Medication Choice Decision aid cards’ with information about medicines allowing the patients to take part in decision of the treatment agent; Patients’ participation in decision making based on information supplied.	Therapy related and Patient related	Clinicians (were randomized to cater for both intervention and usual care group)	**Training**- Yes; **Assessment**- Yes.	No
Rodin HA et al, 2009 [46]	Economic	Provision of free generic drugs and higher co-payments of brand- name drugs.	Socio- **economic** related	Not applicable	Not applicable	No
Sacco WP et al, 2009 [47]	Multifaceted *[Educational + Behavioural+ Affective]*	Telephone coaching sessions guided by checklist, with an intention to: improve knowledge and understanding of how to manage diabetes; enhance self- efficacy; motivate and coach for effective self-care behaviour; provide social support; address goal setting and working to work towards the success of the goal. [*Specific medical advice was not given by the coaches*]	Patient related	'*Coaches*' (paraprofessional:: undergraduates in psychology)	**Training**- Yes; **Assessment**- Yes(?) (Coaches first attended a diabetes education course; then were **trained and supervised by a licensed clinical psychologist** & consulted with members of diabetes health care team if needed.)	
Thoolen BJ et al, 2009 [48]	Multifaceted *[Educational+ Behavioural+ Affective]*	2 individual & 4 group sessions over 12 weeks by registered nurses, done to: Discuss experience with diabetes; Goal/s setting; Recognizing conditions for and barriers to goal attainment; Generating strategies for problem solving; Formulating actions in the form of concrete and proactive 5- step action plan. Prior consideration on how they are going to evaluate the progress (nurse acting as coach facilitating group interaction and practice with the proactive skills), and Evaluation of the progress and plans.	Patient related	Registered nurses	**Training:** Yes; **Assessment**: Not specified	No
Adepu R, Ari SM, 2010 [49]	Educational	Education regarding disease, medication, diet and lifestyle modification at baseline and at each follow up (conducted for a period of 3 months with an interval of 30 days between follow ups).	Condition related and Therapy related	Not specified	Not specified	Yes
Bogner HR, de Vries HF, 2010 [50]	Multifaceted *[Educational+ Behavioural+ Affective]*	The key components of the intervention were (1) Provision of an individualized program to improve adherence to OHA and antidepressants that recognizes patients’ social and cultural context and (2) Integration of T2D treatment with depression management. Integrated care manager provided the care in coordination with physician and played an intermediary role in promoting adherence to medicines. The care manager offered guideline based treatment recommendations, monitored adherence, clinical status, and provided appropriate follow-up.	Condition related, Therapy related, Patient related and **Socio**- economic related	*'Integrated care manager'* (a master’s level research coordinator trained as an integrated care manager)	**Training**- Yes; **Assessment**- Yes	Yes
Borges APDS et al, 2010 [51]	Multifaceted (?) *[Educational + Behavioural (*?*)]*	Standard care and monthly follow up by a single clinical pharmacist, who performed the data collection and monitoring based on method of pharmaceutical care developed by Hepler and Strand; Intervention plan developed according to the data collected. The pharmacist’s performed verbal and written orientations related to the control of the disease, compliance to therapy, appropriate nutrition and correct use of drugs including the method of insulin application; Provision of patient referrals (to specialist) as required.	Condition related and Therapy related	Clinical pharmacists	Not specified	Yes
Castillo A et al, 2010 [52]	Provider level intervention followed by intervention to patients by the providers. Intervention to providers: Multifaceted *[Educational+ Behavioural]* Intervention to patients: Multifaceted *[Educational + Behavioural + Affective]*	DEEP had 2 components that applied participatory techniques and principles of adult education: (1) The Training of Trainers (TOT), a 20-hour workshop that prepares CHWs to implement the educational curriculum for community residents, and (2) The Diabetes Education Program, a series of educational sessions that empower persons living with or at risk of diabetes to address their self-care needs, by increasing diabetes knowledge, developing self-management skills, and facilitating behavioural change. Educational sessions were conducted by the CHW trained (as above) which consisted of: 2-hour sessions scheduled every week for 10 weeks and were led by a team of 2 CHWs (facilitator &assistant), in a class of 10–15 participants, including family and friends. Whenever possible, participants without regular medical care were referred to community clinics, private doctors, or other community resources.	Condition related and **Socio**- economic related	Community health care workers (CHWs)	**Training**-Yes (DEEP is all about training the CHW and then, these trained CHW were involved in care of diabetic patients); **Assessment**-Not specified.	Yes
Cinar FI et al, 2010 [53]	Multifaceted *[Educational + Behavioural+ Affective]*	Phone interviews: (1) Diabetes education in the 1st call (average 30 minute session). Education was about the nature & risk factors of the disease, diet, exercise, drug therapy, hypoglycaemia and hyperglycaemia management; Problems identified and education about these problems was given to the patients individually by the researcher. Patients were informed about the provision of assistance from a research nurse, a dietician and doctor if required. Relatives of patients were also included into the training, if possible. (2) Follow up on patients by telephone (once a week for the 1st month and once every 2 weeks for 2 months, 8 calls/patient on an average). Problem (if any) were detected and the patients were notified about the problem, with a solution during the follow up or as appropriate.	Condition related, Therapy related, Patient related and **Socio**- economic	Nurse	Not specified	Yes
Gonzalez JS et al, 2010 [54]	Multifaceted *[Educational + Behavioural + Affective]*	1 visit with nurse diabetes educator, 2 with dietician &10–12 sessions of CBT- AD. Session started with focus on adherence to medical recommendations, followed by other sessions which were informational, problem- solving, and cognitive behavioural steps that targeted a range of self- care behaviours. Patients were encouraged to focus on positive reasons for being adherent when engaging in such behaviour. Goal setting and moving towards the goal—the goals were changed as required. CBT- AD formed the core component.	Patient related	Nurses, dietician and mental health specialists (?). (Note: who delivered the CBT is not clear.)	Not specified	Yes
Tang TS et al, 2010 [55]	Multifaceted *[Educational + Behavioural + Affective]*	The patient centred sessions conducted by experts that focused on: Reflecting on relevant self- management experience; discussing emotions and feelings; engaging in problem- solving; addressing questions about diabetes and its care and behavioural goal- setting.	Patient related and Condition related	*Sessions were co-facilitated by a 'certified diabetes educator' and a 'clinical psychologist*.'	Not specified	No
Wolever RQ et al, 2010 [56]	Multifaceted *[Educational + Behavioural + Affective]*	An initial telephone session with their coach within 2 weeks of the baseline visit, followed by a 30-minute coaching sessions by telephone (8 weekly calls, 4 biweekly calls, and a final call 1 month later) for total of 14 sessions. These calls focused on: identifying problems, assessing the vision of health as perceived by the patients, assessing what was important to them and how well they were managing the disease, setting goals, getting directions from coaches. Provision of binder of educational materials to the patients at the initial assessment visit.	Patient related, Condition related and Therapy related	‘*Coaches’*	**Training**- Yes; **Assessment**- Not specified.	Yes
Zhang Y et al, 2010 [57]	Economic	The impact of changes to the then existing medical insurance plan to the Medicare Part D Program, with different drug coverages schemes that assisted the patients with cost of the medicines as per the scheme.	Socio- **economic** related	Not applicable	Not applicable	Yes
Gracia- Huidobro D et al, 2011 [58]	Multifaceted *[Educational + Affective]*	Family oriented intervention (meaning incorporating family members in care) consisted of: 2 family meetings or home visits; 1 individual counselling session; 1 counselling session with family; 1 multifamily educational session.	Patient related and **Socio**-economic related	‘*Providers of the intervention clinic*’	**Training**- Yes; **Assessment**- Yes	No
Khan MA et al, 2011 [59]	Educational	Multimedia education: Patients viewed a computer multimedia program (Living Well with Diabetes) in the waiting-room setting. The program content included an introduction to diabetes, blood glucose management, oral medications and insulin, nutrition and physical activity, depression and stress, oral hygiene, and the prevention of complications. Each lesson targeted a specific self-care objective.	Condition related	Not applicable	Not applicable	No
Mehuys E et al, 2011 [60]	Multifaceted *[Educational+ Behavioural+ Affective]*	Community pharmacist delivered: (i) education about T2D &its complications; (ii) education about the correct use of OHAs (timing in relation to food); (iii) facilitation of medication adherence (by counselling); (iv) healthy lifestyle education (diet, physical exercise & smoking cessation); and (v) reminders about annual eye and foot examinations.	Condition related and Therapy related	Community Pharmacists	**Training**- Yes; **Assessment**- Not specified.	No
Mitchell B et al, 2011 [61]	Multifaceted *[Education+ Behavioural + Affective]*	On their 1st Diabetes Medication Assistance Service (DMAS) visit, patients were given a blood glucose meter, instructed on its use, and asked to take measurements. During the next 4 DMAS visits, the pharmacists downloaded the patient’s BG readings and generated printouts and charts. Based on this report, pharmacist &patient discussed on areas of inadequate glycaemic control, and pharmacist delivered appropriate *self-management support interventions* (SMSIs). Any specific concerns the patient had about their T2DM was taken into account. Goals to be achieved by the next visit were negotiated and documented.	Condition related and Patient related	Community Pharmacists	**Training**-Yes; **Assessment**- Not specified.	Yes
Piette JD et al, 2011 [62]	Multifaceted *[Educational+ Behavioural + Affective]*	12-month telephone delivered CBT program, which included an initial intensive phase of 12 weekly sessions followed by 9 monthly booster. Initial focus on depressive symptoms with gradual introduction of concepts related to links among depression, physical activity, & diabetes outcomes by nurse counsellors–the sessions were based on weekly manual. During each session, nurses monitored patients’ depressive symptoms, and their activity levels. Communications with health care providers when required and warning sent to PCPs in the event that the patient reported: suicidal ideation, discontinuing antidepressant medication on their own, persistent elevated depressive symptoms, or a need for a prescription refill.	Patient related, Condition related; and, Health system related (?)	Nurse	**Training**- Yes; **Assessment**- Yes.	No
Ramanath KV, Santhosh YL et al, 2011 [63]	Educational	Provision of educational materials PIL (Patient information leaflet) and formal counselling from the clinical pharmacist	Condition related and Therapy related	Clinical pharmacist	Not specified	Yes
Shetty AS et al, 2011 [64]	Behavioural	SMS reminders once in every 3 days to follow diabetic regimen [regimen of dietary modification, physical activity and drug schedules]	Patient related	Not specified	Not specified	No
Smith SM et al, 2011 [65]	Multifaceted *[Educational+ Affective]*	The intervention was a peer- support intervention consisting of the following major elements: The recruitment and training of peer supporters, based on protocol in the participating practices; Nine group meetings led by peer supporters in participants’ own general practice. Each meeting had a suggested theme related to the diabetes care. At the end of each session the group fed back questions to the research team who compiled written answers based on feedback, which was combined and sent to the groups for next session. Peer supporters besides the meetings were also in contact with the participants via telephone calls and letters. Formal structures to ensure retention and support of the peer supporters.	Patient related and **Socio**- economic	Peer supporter	**Training-** Yes; **Assessment**- Yes.	No
Wakefield BJ et al, 2011 [66]	Multifaceted *[Educational + Behavioural]*	The intervention made use of a home-telehealth device which allowed data transmission between patient's home and the study centre. Intervention patients entered BP and BG measurements and responded to standardized questions. Patients then received appropriate automated responses depending on how they answered the device prompt: correct responses were reinforced and incorrect responses were reviewed and explained. The device also allowed individualized messages to be transmitted to subjects. Each day, the study nurse reviewed the responses and determined whether the subject needed follow- up, that is additional health information, increased monitoring, compliance strategies, problem resolution facilitation, or contact with physician.	Condition related and Patient related	Nurses	Not specified	No
Walker EA et al, 2011 [67]	Multifaceted *[Educational + Behavioural]*	Tailored telephone calls to the patients [up to 10 calls at 4- to 6-week intervals for 1 year] by health educators that focused on diabetes medication adherence and on lifestyle changes through healthy eating and physical activity. The call contents were guided by a manual. The following main elements were considered during the intervention: Problem solving, Goal setting, Communication skills, and Preplanning for medical visits. Participants received selected high-quality self-management materials by mail, and were prompted by health educators to use these materials.	Patient related, Condition related, and Therapy related	Health educators	**Training**-Yes; **Assessment**-Yes(?) (the health educators were trained and supervised by certified diabetes educator nurse)	Yes
Barron JJ et al, 2012 [68]	Study 1: Economic; Study 2: Educational	Study 1: Evaluated the impact of a 50% reduction in co-payments, on a prior approval (PA) basis. Study 2: Provision of waived co-payments for participation in a mandatory Telephonic Diabetes Education and Support (TDES) initiative.	Socio- economic related and Patient related	Not applicable	Not applicable	Study 1: No; Study 2: No
Bogner HR et al, 2012 [69]	Multifaceted *[Educational + Behavioural+ Affective]*	The intervention consisted of 3, 30-minute in-person sessions (at baseline, 6 weeks, and 12 weeks) and 2, 15-minute telephone-monitoring contacts between the patient and integrated care manager to offer education and guideline-based treatment recommendations to patients and to monitor adherence and clinical status. The key components were: (1) the provision of an individualized program to improve adherence to antidepressants and OHAs that recognizes patients’ social and cultural context, and (2) the integration of depression treatment with T2D management.	Patient related, Therapy related, Condition related and **Socio**- economic	Research coordinators (1 master’s level and 1 bachelor’s level) who were trained as 'integrated care managers'	**Training**- Yes; **Assessment**- Not specified.	Yes
Brennan TA et al, 2012 [70]	Multifaceted *[Educational + Behavioural]*	For adherence enforcement: Follow-up call from a pharmacist, from either the retail setting or the mail-order pharmacy to the patients who were late in refilling an anti-diabetic medication, to discuss non- adherence and offer to refill mail prescriptions; the pharmacist also checked the requirement of statins and ARB for the patient and if considered required communicated the need with the patient and with his consent with the physician, and delivered the physician’s decision to the patient; and a follow-up call from pharmacy advisor team 30 days after initial call.	Patient related and Therapy related	Pharmacist	**Training**- Yes; **Assessment**- Not specified.	Yes
Choi SE, Rush EB, 2012 [71]	Educational	2 sessions of culturally tailored diabetes self- management education, led by an experienced bilingual family nurse practitioner. The contents of sessions were those considered essential by the ADA and the National Diabetes Education program.	Condition related, Socio- economic related, and Patient related	Family nurse practitioner	Not specified	No
Farmer A et al, 2012 [72]	Multifaceted *[Educational+ Behavioural + Affective]*	Medication dispensed in medication monitoring device. Consultation with clinic nurse at follow up visits, consultation had the following components: (1). the motivational component, where the nurse explored patients`beliefs relevant to their intention to take medication regularly as prescribed (eg. perceived benefits and harms of taking medicines, views of other people who were important to them and factors that may facilitate or inhibit day to day medicine taking as prescribed.)→tailored information provided verbally and non-verbally to reinforce positive beliefs and facilitation for problem solving around negative beliefs. (2). action planning component, the nurse asked patients to generate and write down the exact circumstances in which they would take their medication (using an “if-then” formulation to elicit where, when and how this would occur).	Patient related	Clinic nurse	**Training-** Yes; **Assessment-** Yes.	Yes
Kroese FM et al, 2012 [73]	Multifaceted *[Affective+ Behavioural]*	The study evaluated the impact of booster sessions to ‘Beyond good Intention’ intervention*. The patients were provided with the intervention, followed by booster sessions and evaluation of the impact of booster session was conducted. Booster sessions comprised of 3 group sessions scheduled 1, 3 & 6 months after the end of the initial phase. After the initial phase, the patients were randomly divided in two types of booster conditions, the ‘how’ versus ‘why’ condition. The content of the ‘**how’-condition** was identical to the initial phase of the intervention, where the participants were instructed to step-by-step transform their self-management goals to concrete sub goals (e.g. ‘How are you going to get more exercise’). In the ‘why’-groups, patients were challenged to consider their self-management goals in view of their higher-order overarching goals (e.g. ‘Why are you going to get more exercise’).	Patient related	*'Instructed trainers’* (Not clear, states: *At one point for the booster sessions*, *it is written that the sessions were led by 'instructed trainers')*	Not specified	No
Mellitus Janice C-M et al, 2012 [74]	Multifaceted *[Educational + Affective]*	Patient attended Education sessions on focused on 7 areas of self-care management, held once a week for 2 hrs over 6 weeks. The sessions were emotion focused & integrated spiritual coping. Culturally targeted written materials, videotapes, and presentations by racially concordant health-care providers and research staff were also provided.	Condition related	‘*the sessions were led by the Health care providers*’ *and 'clerics’*	Not specified	Yes
Odegard PS, Christensen DB, 2012 [75]	Multifaceted *[Educational+ Behavioural + Affective]*	Pharmacist phone call as follow up to a missed diabetes prescription refill. During the call, pharmacists asked whether patients had exhausted their medication supply, inquired the reason for the late refill, identified &addressed adherence challenges, provided customized diabetes adherence education & encouragement, and developed a self-management action plan. A scheduled follow- up call between 1 week &1 month following intervention, in order to assess needs, confirm problem resolution, and provide self-management encouragement.	Patient related	Pharmacists	**Training**- Yes; **Assessment**- Yes (?) (at the beginning of the intervention to review all was set to go)	Yes
Ramanath KV et al, 2012 [76]	Multifaceted *[Educational + Behavioural]*	Counselling (about disease drug and their management) to the patients; Patient information leaflet (at baseline); Diary cards as a medication adherence reminder (to both groups).	Condition related	Clinical pharmacists	Not specified	Yes
Vervloet M et al, 2012 [77]	Behavioural	All patients received their medication in the RTMM medication dispenser and had their medication use registered in real time. For the intervention group, SMS reminders (in response to unopened medication dispenser within an agreed time interval) were sent to patient but not in the control group.	Patient related	Not specified	Not specified	Yes
Zolfaghari M et al, 2012 [78]	Multifaceted *[Educational + Behavioural]*	Initial 3 days of diabetes self- care education SMS group: Information about various self- management behaviours & stress management via SMS; 6 SMS per week [72 SMS/patient in total]. Telephone group: Telephone counselling on nature of disease and self- management behaviours and inquiry of problems &suggestions on how to solve them; Contact time: twice a week for the 1st month & then weekly for 2nd& 3rdmonth.	Condition related and Patient related	“*Researcher”*	Not specified	No

*Referenced as: Thoolen B, De Ridder D, Bensing J et al. Beyond good intentions: the development and evaluation of a proactive self-management course for patients recently diagnosed with type 2 diabetes. Health Educ Res 2008; 23: 53–61.

(?): Not clearly stated in the study; the authors of the review consider it most possible.

The majority (n = 46) of the interventions had an ‘educational’ component [27–30, 32–45, 47–56, 58–63, 65–72, 74–76, 78]. Thirty eight had a ‘behavioural’ component [27, 29–43, 47, 48, 50, 52–56, 60–62, 64, 67, 69, 70, 72–78], and 23 interventions [28, 35, 37, 40, 41, 47, 48, 50, 52–56, 58, 60–62, 65, 66, 69, 72, 73, 75] had an ‘affective’ component.

#### 3.2. WHO dimensions of non-adherence factors addressed by the interventions

Most interventions addressed patient-related (n = 35) [27–29, 31, 33–35, 37, 38, 40, 41, 43, 45, 47, 48, 50, 53–56, 58, 61, 62, 64–73, 75, 77, 78], condition-related (n = 31) [27–29, 35–37, 39–44, 49–53, 55, 56, 59–63, 66, 67, 69, 71, 74, 76, 78], and therapy-related (n = 20) [28, 29, 33–37, 39, 44, 45, 49–51, 53, 56, 60, 63, 67, 69, 70] factors. Only a few took into consideration the socio-economic (n = 13) [37, 40, 41, 46, 50, 52, 53, 57, 58, 65, 68, 69, 71] and health care system-related factors (n = 5) [28, 30–32, 62]. While none covered all five, thirteen studies [28, 29, 35, 37, 40, 41, 50, 53, 56, 62, 67, 69, 71] addressed three or four factors. The remainder either addressed one (n = 18) [30, 32, 38, 42, 46–48, 54, 57, 59, 64, 68, 72–77] or two (n = 21) [27, 31, 33, 34, 36, 39, 43–45, 49, 51, 52, 55, 58, 60, 61, 63, 65, 66, 70, 78] factors (Table 1). The details of the ‘telephonic diabetes education and support’ constituting one intervention were not provided, hence it was not possible to predict the factors that were addressed by the education program, although a general assumption could be made that multiple factors, patient related, condition related and therapy related factors, could have been included [68].

#### 3.3. Interventionist(s)

Of those studies where the interventionist(s) was clearly identified, pharmacist(s) [28, 33, 34, 51, 60, 61, 63, 70, 75, 76] and nurse(s) [29, 36, 38, 42, 48, 53, 62, 66, 71, 72, 78] were involved in delivering the intervention in ten and eleven studies, respectively (Table 1). Community health workers (n = 2) [41, 52], peer supporters (n = 1) [65], bachelor level research assistants (n = 2) [31, 69], masters level research coordinators (n = 2) [50, 69], general practitioners/ clinicians/ physicians (n = 2) [30, 45] were involved in the other interventions. The educators involved, were either professional educators, for example diabetes educators (n = 3) [40, 44, 55], and health educators (n = 1) [67] or were behavioural coaches (n = 2) [47, 56]. Several authors reported the interventionist(s) simply as ‘investigator(s)’ [27, 39] or ‘researcher(s)’ [35]. A few studies did not specify who delivered the intervention [37, 49, 58, 64, 73], and in four studies, an interventionist was not applicable as the studies evaluated the impact of value based insurance designs or Medicare part D program [46, 57, 68]; or the intervention was a multimedia program [59].

#### 3.4. Training to the interventionist and assessment of intervention delivery

Interventionists were trained for intervention delivery in 36.5% of the studies (n = 19) [35, 38, 41, 45, 47, 48, 50, 52, 56, 58, 60–62, 65, 67, 69, 70, 72, 75] and in only 11.5% of the studies was the delivery of the intervention assessed (n = 6) [45, 50, 58, 62, 65, 72] in order to determine how the intervention was delivered (Table 1).

#### 3.5. Characteristics of interventions reporting improved medication adherence

Approximately 41.5% (n = 22) of the studies [31, 32, 36, 44, 49–54, 56, 57, 61, 63, 67, 69, 70, 72, 74–77] reported improvements in medication adherence. The interventions reporting improved medication adherence were mostly multifaceted (n = 16) [32, 36, 50–54, 56, 61, 67, 69, 70, 72, 74–76], followed by educational (n = 3) [44, 49, 63], behavioural (n = 2) [31, 77], and economic (n = 1) [57]. In addition, the non-adherence factors addressed by the interventions had a similar trend. Five studies [50, 53, 56, 67, 69] addressed either three (n = 2) [56, 67] or four (n = 3) [50, 53, 69] factors, while nine [31, 36, 44, 49, 51, 52, 61, 63, 70] and eight [32, 54, 57, 72, 74–77] studies addressed either two or one factor, respectively.

The interventionists involved in delivering these interventions varied. Pharmacists and nurses were involved in six [51, 61, 63, 70, 75, 76] and three [36, 53, 72] interventions, respectively. A nurse was also involved in another, along with a dietician and another professional [54]. Student level researchers were involved in three studies [31, 50, 69] and in two, they were referred to as ‘integrated care managers’ [50, 69]. Two studies mentioned respectively ‘health care professionals’ [44] and health care providers [74] were involved in leading the intervention sessions, without specifying who they were. “Coaches” [56] and Community Health Workers (CHWs) [52] were interventionists in other studies. Three studies did not specify who the interventionist was [49, 57, 77], and in two an interventionist was not required as these studies either evaluated the effect of implementing the Medicare part D program on anti-diabetic medication adherence [57], or the patients were delivered their OHAs in Real Time Medication Monitoring (RTMM) medication dispensers, which helped in registering their medication real time [77].

The interventionists were trained for the delivery of the study intervention in eight studies [50, 52, 61, 67, 69, 70, 72, 75]. Two studies [50, 72] assessed the delivery of the intervention process, while two others mentioned providing supervision of [67], and consultation with [75] the interventionists during the intervention delivery.

#### 3.6. Characteristics of interventions reporting improvement in HbA1c

HbA1c was an outcome measure in 64.1% of the studies (n = 34) [27–29, 31, 33–41, 43, 47, 50–56, 58–60, 62, 64–67, 69, 71, 74, 78]; 16 of which [33, 36, 39, 50–55, 58, 60, 64, 66, 67, 69, 71] reported a significant impact on HbA1c. More than 80% of these interventions were multifaceted, and more than 85% (n = 14) addressed two or more WHO non-adherence factors.

#### 3.7. Characteristics of interventions reporting improvement in both medication adherence and HbA1c

Out of the 34 studies that assessed the impact of interventions on both medication adherence and HbA1c, nine [36, 50–54, 56, 67, 69] reported a positive impact on both medication adherence and HbA1c. However, in one study, the improvements in HbA1c were seen in patients with HbA1c>7%, rather than all participating patients [56]. The interventions were all multifaceted. In one study, the educational component was the primary component; however, it appeared that there was also a behavioural component, though it cannot be stated with certainty without obtaining more detailed information about the program [51]. Three studies [50, 53, 69] addressed condition-related, patient-related, therapy-related and socio-economic factors. Two others [56, 67] targeted the former three factors. One [54] was focused only on patient-related factors. Two addressed condition and therapy-related factors [36, 51], and one [52] **addressed four: patient, condition, therapy and socio-economic-related factors**. None addressed the health care system related factors.

Two studies [50, 69] included patients who had diabetes and depression and involved integrated care management for diabetes and depression. Both of these studies, published by the same authors, employed the same intervention strategy. The intervention was multifaceted, delivered by trained ‘integrated care managers’ and addressed patients’ individual, social and cultural needs, and was designed to improve medication adherence, glycaemic control and depression outcomes.

Two studies consisted of telephone-based interventions to improve diabetes control [53, 67]. One involved telephone calls conducted by nurses to deliver education about of diet, exercise and medication adherence [53]. In the other, participants received telephone calls from health educators primarily about diabetes medication adherence [67].

Other interventions consisted of SMS delivered by nurses to educate the patients and aid in reinforcement of diet, exercise and medication adjustment [36]; a diabetes education program delivered by community health care workers [52]; pharmaceutical care program [51]; integrated health coaching [56] and a cognitive behavioural therapy for adherence and depression [54].

#### 3.8. Major elements in the intervention processes and changes over time

Overall, interventions have been refined over time, both in terms of their design, and delivery. The majority of the interventions were multifaceted, with increasing incorporation of affective components, especially for interventions implemented since 2010. Approximately 35.7% of the interventions published prior to 2010 included an affective component compared to 42.1% after 2010.

Educating patients, individually or in groups, on various behavioural and medicine related issues has remained one of the most widely used strategies to promote adherence over the past 13 years. Similarly, using telephones has remained a popular strategy for delivering interventions and conducting follow-ups. However, more recently, mobile phones have been used to deliver educational messages as well as send text reminders as behavioural prompts (eg in collecting refills and taking medications) [36, 64, 78].

Interventions published, particularly after 2005, were more intensive and complex. The interventions not only addressed the patients’ knowledge gap and problems related to self-management practices, but also incorporated a range of other pertinent issues, for example ‘cognitive improvement’ [35], ‘cultural issues’ [37, 40], ‘inclusion of support person’ [37], ‘stress and stress management’ [37] and ‘self-efficacy’ [37, 47]. Concepts such as ‘integrated health coaching’ [56], ‘cognitive behavioural therapy’ [54, 62] were also implemented and evaluated. Goal setting as an intervention strategy was seen to emerge in the studies published after 2007 [43, 48, 56, 67]. Thus, over time the interventions have become more patient-centred attempting to address specific patient needs with focus on adding more affective approaches.

Use of technology for designing interventions was reported more in the studies published after 2005. Such, technology driven interventions, varied from simple strategies, for eg. using a cell phone or an internet to input blood glucose data and receive feedback [36], to the use of complex computer programs, such as *NICHE technology* [38] and *Well Doc’s Proprietory Management Software* System [39] for diabetes management.

Three studies, published since 2009 [46, 57, 68] have addressed the economic component, which had not been addressed previously.

Although interventions have become more complex in recent years, more ‘straightforward’ strategies such as delivering education materials, providing routine education sessions, and displaying multimedia programs in waiting room settings, are still used in some interventions. Moreover, the complex interventions have involved the addition of more patient oriented components to these ‘simple’ strategies, and these have proven slightly more successful than the ‘simpler’ ones.

## Discussion

There has been a significant increase in the number of studies implementing and evaluating interventions aimed at promoting adherence to anti-diabetic medications, with the majority being conducted in the USA. Overall, the systematic review identified a range of intervention techniques which have been employed in an effort to improve self–care behaviours including adherence to anti-diabetic medications, in patients with T2D. The interventions were primarily directed at patients and ranged from simple educational interventions, including supply of educational materials, to complex interventions which took into consideration patients’ attitudes, practices and preferences in addition to educating and aiding them to adhere to therapy. In fact, a majority of the interventions were multifaceted, consisting of a combination of more than one educational, behavioural or affective strategy. The interventions were delivered mainly by nurses and pharmacists; only a few studies reported having trained the interventionist on delivering the intervention, and even fewer reported assessing the delivery of the intervention by the interventionists.

Only a few of the interventions reviewed reported a significantly positive impact on adherence to anti-diabetic medication, and even fewer had a positive impact on both medication adherence and HbA1c levels, a parameter assessed in most studies as the measure of glycaemic control. Most of these successful interventions were multifaceted, employing a combination of strategies. However, interventions with comparable approaches showed varying results, and made it very difficult to deduce the effective intervention components. It was outside the scope of this review to investigate the components and delivery of the interventions in-depth, to determine whether the interventions were developed appropriately and/or adequately delivered by the most suitable healthcare professional. This level of investigation requires access to process implementation data which may not have been collected by the original researchers.

Combination strategies have been recognized as more likely to enhance outcomes and reduce costs for patients with T2D [79]. Whereas this aspect is undoubtedly supported by the findings in the review, a few interventions which consisted of an educational, behavioural or economic component only, have also had an impact on anti-diabetic medication adherence. Overall, educational content was the most common component of the interventions. This indicates the importance of educating patients about their condition and its therapy as a first step in increasing awareness about the importance of adherence to treatment and motivating patients to take their medications to ensure improved disease management and better health outcomes. Moreover, educating patients through the provision of written materials, electronic media, and verbally, could perhaps be regarded as one of the simplest and most efficient strategies to implement when the goal is to promote patients’ adherence to their therapy. However, effective education and determining that patients have understood the information and that the information has not only increased knowledge but resulted in a behavioural change, presents a greater challenge to researchers, clinicians and healthcare providers involved in delivering interventions to patients with T2D.

Behavioural strategies, which are increasingly being used in adherence support interventions, range from adherence aids (eg dose administration aids) which address non-intentional adherence, to motivational interviewing and other more complex strategies, which aim to address several factors impacting patients’ medication taking behaviour. Non-adherence may be due to a multitude of factors, and a combination of educational and behavioural strategies provide a greater likelihood of addressing the factors that impede adherence in a broader group of patients. Additionally, diabetes management involves management of lifestyle issues, and therefore, it becomes quite important for patients to adapt to the changing circumstances. Behavioural component(s) would aid the patient in the process of adaptation, and educational strategies will provide the information needed to facilitate the behavioural changes. Thus, it is not surprising that most of the interventions designed over the last few years have consisted of both educational and behavioural components.

Affective components were employed from the earlier years although affective components have become more common in the later years, and are present in most of the interventions. Newer approaches such as inclusion of ‘peer supporters’ as interventionists, addressing cultural issues and ethnic beliefs, adding novel techniques like integrated health coaching, and addressing cognitive issues in the more recent years, has added to the ‘affective’ component, while also making the newer interventions more patient-centred. While simple individual counselling or an education session could be regarded as being focussed on a single patient, the impact would however depend upon the extent to which an individual’s psychosocial needs are taken into consideration. Psychosocial issues, such as attitudes about illness, affect, mood, diabetes related quality of life, resources, diabetes-related distress, and cognitive abilities have been recognised in recent years as important predictors of overall diabetes management [80]. Furthermore, from a global perspective, cultural issues and societal orientation have also been found to influence diabetes management [81, 82]. Issues such as these have been recognised as important predictors of adherence and diabetes management and been included in interventions, thereby increasing the ‘affective components’ in recent years.

Interactive technology is increasingly being explored for health promotion [83] and was also employed in adherence promoting interventions identified in this review, in order to deliver educational, behavioural and affective components, either singly or in combination. In its simplest approach, interactive technology was used to educate, remind, or provide advice to patients. Other examples of interactive technology used were the Well-doc System, NICHE technology, tele-health devices, and RTMM technology. These technologies enabled the collection and transfer of patient specific data/ information across to different professionals/ personnel, who could then deliver the tailored feedback and reminders to the patients. The increasing advancement in technology and benefits that can be received particularly with regard to patients being in contact with their care providers from home is an appealing prospect. Furthermore, technology driven interventions could have a greater reach, better adoption and implementation, and increased sustainability; thus having a greater positive heath impact [84]. However, more research is needed to establish the sustained effects of such technologies and to evaluate how such technologies can really be useful in the short and long term in promoting adherence to medications.

Human behaviour in itself is a complex phenomenon. It is therefore more likely that any intervention designed to influence human behaviour, such as modifying medication adherence or other self-care behaviours in patients with T2D, would be more successful if multiple factors that intertwine to aid the change in complex human behaviour are addressed. Combined interventions comprise of different components, which may act both independently and inter-dependently [85], to address the change(s) desired, and may be more effective than using a single component in isolation. However, complexity involved in designing, implementation, evaluating and replicating ‘combined intervention(s)’, often complicates the practicalities and interventions involving a single component may be preferred as they are easier to design, implement and replicate, and often times are successful in influencing a behaviour change.

It is recommended that the diabetes services be led by the needs of patients [80]. Hence, it is logical that interventions designed for a behaviour change are guided by patients’ need. Individualizing or tailoring strategies for behaviour change to the needs of the individual patients is more effective than a ‘generic form’ of any strategy; for example, tailoring health behaviour change messages have been found more effective than generic communications [86]. In tailoring an intervention, the strategies tend to be more patient centred. More than half of the interventions included in this review considered the individual patient’s circumstance(s) while delivering the intervention; however, the extent of individualization varied. Each component (educational, behavioural, affective or economic component) needs to be individualised, and rendered more patient centred. Williams et al have also emphasised the need for ‘tailored interventions’ to promote medication adherence in patients with T2D [24].

The factors affecting adherence to anti-diabetic medications have been inconsistently reported [87]. However, WHO has identified five factors that interact to influence patients' adherence behaviour [5]. WHO patient-related, condition-related, and therapy-related factors contributing to non-adherence were addressed by most of the interventions identified in this review. The socio-economic factors were addressed by fewer interventions and hardly any covered the health-care system related factors. However, there was a growing trend in the later years in incorporating socio-economic factors into the intervention designs. The impact of socio-economic factors and health care system related factors on medication adherence has been established particularly in cases of chronic diseases [81, 88], though their influences have been found to be 'inconsistent' [5]. As the evidence increases, it is likely that more interventions will include strategies to address socio-economic and health care system factors impacting adherence, as was seen in the more recent interventions identified in this review.

Financial or economic factors could become a major issue for patients with chronic diseases. Management of long term conditions such as diabetes and the consequences of poorly controlled diabetes could have an astounding economic impact on the patient and on each country globally [89, 90]. Although the magnitude of the impact is more likely to depend on the economic status of the patients, reduced out of pocket expenses have been identified as a factor that could improve medication adherence [91]. Only a few studies have looked into this aspect, with three identified as part of this review. One evaluated the impact of US ‘Medicare Part D’ program on adherence, another assessed the impact of generic substitution in reducing costs, and the third focused on the impact of a value based insurance program. Whereas the former was successful in improving medication adherence to anti-diabetic medications, the latter two were not. Nothing conclusive can be drawn about how cost impacts adherence from these studies. However, expenses in terms of medication taking, diabetes management, and the consequences of not adhering to medications could have a staggering economic impact [90]. More studies are therefore needed to explore the impact of a range of economic interventions. As mentioned, health-care system related factors, including healthcare professionals, were the least addressed WHO adherence factors in the interventions included in this review. Although health care providers like nurses, pharmacists and sometimes clinicians were involved in delivering the intervention, whether they had sufficient training in the matters relating to patient adherence behaviour was unknown. Their involvement could largely be observed as a supplier of patient-related, therapy-related and condition-related information and have been classified accordingly. Health care systems globally are intricate; effectively addressing health care system during trials could pose practical difficulties, which could be a reason for the small number of interventions addressing health care system related factors. However, as they cover quiet a significant expanse in patients' overall health care behaviour, it is necessary that these factors be explored and addressed effectively when designing future interventions for patients with T2D.

In summary, while most of the interventions addressed multiple factors, none were able to cover all five WHO factors. An ideal intervention would be one that explores and addresses all interacting factors through a tailored strategy to bring about a change in patient medication adherence [87]. However, nothing conclusive could be drawn from the studies included in this review, as to the successful intervention, as the majority were found to address only one or two of these interacting factors. The factors and the extent of their influence could vary between people, and is likely to be addressed by tailoring of the intervention. Further research also needs to focus on the 'extent' of the influence of the different factors. Ample consideration is needed in terms of how such interacting factors could be effectively addressed and incorporated to improve medication adherence.

## Conclusion

Multifaceted interventions appear to be a more effective approach in improving medication adherence and glycaemic target in patients with T2D than individual interventions, as they provide a range of strategies to address a number of factors that may impact an individuals' adherence to their medications. Educational component formed the most widely used component in the interventions, followed by behavioural and affective components. While educational components have remained popular throughout, affective components have become more common in the later years. Nonetheless, it is extremely difficult to conclusively state which strategies are more effective, because interventions with similar components have proved to be successful in some studies and unsuccessful in others. Additionally the variation observed in research designs, and methods of outcome assessments, together with differences in patient characteristics prevented a detailed comparison of findings or a meta-analysis.

Patient-related, therapy-related and condition-related factors were addressed by most of the interventions. Health care system related factors were least addressed. While 'social' factors are increasingly being addressed, the focus on the 'economic' aspect is still lagging behind. In addition to effectively addressing all these factors, future research needs to focus on exploring the extent to which such factors influence an individuals' medication taking behaviour throughout the medication taking journey.

Overall, future research investigating development and evaluation of effective and sustainable adherence promoting interventions needs to include standardized methods and tools, and prioritize the needs and therefore tailor interventions to the needs of individuals.

## Limitations of the Review

The diverse nature of the studies included in the review presents an important limitation preventing a detailed comparison of the interventions (and a meta-analysis). Moreover, studies included were not selected based on their quality. The review was conducted with a broad approach, and intended to include all studies addressing anti-diabetic medication adherence in patients with T2D irrespective of their quality. The components of the interventions and their categorization into the WHO factors for each study have been based only on the information provided in the manuscripts. This could have had an influence on those classifications where the interventions were not explained in sufficient detail. However, the authors have done their best to ensure proper identification of intervention components using the information provided.

## Supporting Information

S1 PRISMA Checklist PRISMA Checklist. (DOC)

S1 Fig Literature review search strategy. (DOCX)

S2 Fig PRISMA Flow diagram. (DOC)

S1 Table Review inclusion and exclusion criteria. (DOCX)
